# Effects of syringic acid on the indomethacin-induced gastric ulcer model in rats: in vivo and in silico study

**DOI:** 10.1007/s00210-026-05351-4

**Published:** 2026-05-06

**Authors:** Aslıhan ATASEVER, Fikret ÇELEBİ, Serkan YILDIRIM, İsmail BOLAT, Burak ÇINAR

**Affiliations:** 1 Department of Veterinary Medicine, Çayırlı Vocational High School, Erzincan Binali Yıldırım University, Erzincan, Turkey; 2 Faculty of Veterinary Medicine, Department of Physiology, Atatürk University, Erzurum, Turkey; 3 Faculty of Veterinary Medicine, Department of Pathology, Atatürk University, Erzurum, Turkey; 4 Faculty of Medicine, Department of Pharmacology, Atatürk University , Erzurum, Turkey; 5 Faculty of Veterinary Medicine, Department of Pathology, Kyrgyzstan-Turkey Manas University, Bishkek, Kyrgyzstan

**Keywords:** Apoptosis, Indomethacin, Inflammation, Oxidative stress, Syringic acid, Gastric ulcer

## Abstract

In this study, the potential protective effects of syringic acid (SA) on gastric tissue were investigated in an indomethacin (INDO)-induced gastric ulcer model. A total of 84 male Sprague–Dawley rats were randomly divided into seven groups. In the in vivo experiments, rats were administered SA at doses of 5, 50, and 100 mg/kg and omeprazole (OMP) at a dose of 5 mg/kg intragastrically (i.g.) for 14 days, and indomethacin (100 mg/kg, i.g.) was administered on the final day. Following INDO administration, the rats were sacrificed under anesthesia, and gastric tissues were carefully excised for further analyses. The collected gastric tissues were subjected to biochemical, histopathological, and immunofluorescence analyses. In addition, in silico analyses were performed to support the INDO-induced gastric ulcer model. Using the licensed Schrödinger Maestro 2025/1 software, the binding properties of INDO to the COX-1 receptor were evaluated through molecular docking, MM-GBSA, and pharmacophore matching analyses. INDO administration was associated with oxidative stress, inflammation, apoptosis, and histopathological damage in gastric tissue. SA treatment appeared to alleviate INDO-induced gastric injury through its antioxidant, anti-inflammatory, and anti-apoptotic properties. SA treatment ameliorated histopathological alterations in ulcerated areas, particularly at doses of 50 and 100 mg/kg, whereas the 5 mg/kg dose did not show a significant protective effect. In addition, in silico analyses suggested that INDO may contribute to ulcer formation by inhibiting COX-1, thereby reducing prostaglandin production in the gastric mucosa. Overall, the findings of this study suggest that SA may reduce oxidative stress, suppress inflammatory responses, and inhibit apoptosis, thereby contributing to the protection of gastric tissue against INDO-induced injury. These results indicate that SA may have therapeutic potential for the prevention of NSAID-induced gastric injury; however, further experimental and clinical studies are needed to confirm these effects and clarify the underlying mechanisms.

## Introduction

Ulcers are defined as disruptions in the mucosal lining of the digestive tract. These disruptions can extend from the muscularis mucosa layer into the submucosa or even deeper layers. They can develop in any part of the gastrointestinal system that is excessively exposed to acid-peptic secretions (Kumar et al. 2014). Gastric ulcer (GU) is a chronic gastrointestinal condition characterized by a high clinical incidence and recurrence rate. Common symptoms include upper abdominal pain, bloating, belching, and acid reflux. In more severe cases, signs such as hematemesis and melena may occur. Complications like gastric perforation, gastrointestinal bleeding, and malignant transformation can also develop, potentially leading to significant impacts on the patient’s health and quality of life (Lau et al. 2011). The healing of gastric ulcers involves a regenerative process characterized by cellular differentiation, proliferation, migration, re-epithelialization, and formation of granulation tissue, as well as angiogenesis and vasculogenesis (Tarnawski and Ahluwalia 2021). The main principle of treatment is to protect the gastric mucosa and suppress acid secretion. Among the most common treatment approaches for gastric ulcers is the inhibition of acid secretion through the use of proton pump inhibitors. Omeprazole (OMP), a proton pump inhibitor, can significantly reduce gastric acid production. It may also lessen the mucosal degradation caused by gastric acid and help alleviate the symptoms of gastric ulcers (Kangwan et al. 2014).

Nonsteroidal anti-inflammatory drugs (NSAIDs), such as indomethacin (INDO), are widely used to relieve pain and reduce fever in inflammatory conditions. However, these drugs are associated with adverse effects like gastrointestinal toxicity. Such effects can lead to complications including gastric ulcers, bleeding, and perforations in the digestive tract (Teichert et al. 2014; Morsy and El-Moselhy 2013) . Ulcers develop as a result of an imbalance between aggressive factors and the body's protective mechanisms (Kumar et al. 2014). Gastric ulcer is a common condition, with contributing factors including gastric acid, pepsin, bile salts, *Helicobacter pylori* infection, alcohol consumption, and the use of NSAIDs (Musumba et al. 2009). NSAIDs can damage the gastric mucosa, thereby playing a role in ulcer formation (Fornai et al. 2011). Among NSAIDs, indomethacin is known to have the highest ulcerogenic potential (Al Asmari et al. 2014). The main mechanisms underlying gastric pathogenesis include cyclooxygenase (COX) inhibition, reduced prostaglandin (PG) synthesis, oxidative stress, and inflammation triggered by indomethacin (Takeuchi 2012).

Phenolic compounds are secondary metabolites commonly found in many plants, fruits, and vegetables. They are biosynthesized through the phenylpropanoid and shikimic acid pathways. Acting as natural antioxidants, these compounds help reduce oxidative stress by scavenging cellular reactive oxygen species (ROS) (Prior et al. 1998). Natural compounds with antioxidant properties are used in the management of various disorders, including cardiovascular diseases, liver damage, cancer, neurodegenerative conditions, and diabetes (Soobrattee et al. 2005). One of the natural compounds in this category is syringic acid (SA). SA is a phenolic compound abundantly found in pumpkin, grapes, olives, spices, red wine, honey, acai palm, and many other plants (Pezzuto 2008; Pacheco-Palencia et al. 2008). It is recognized as a natural antioxidant with a wide range of biological activities, including free radical scavenging, anticancer, anti-inflammatory, antimicrobial, antidiabetic, and cardio-neuroprotective effects. The therapeutic potential of SA is mainly attributed to the presence of methoxy groups on its aromatic ring. It can neutralize free radicals, modulate enzyme activities, and regulate several transcription factors involved in inflammation, angiogenesis, diabetes, and cancer (Cheemanapalli et al. 2018). Moreover, SA is well documented for its antioxidant and anti-inflammatory activities, as well as its antimicrobial and anticancer properties. In addition, SA exhibits anti-adipogenic and antidiabetic effects and has been reported to exert hepatoprotective, neuroprotective, and cardioprotective activities, which have attracted considerable interest in the biomedical field (Muthukumaran et al. 2013; Li et al. 2019; John and Arockiasamy 2020).

Recent studies have reported the protective effects of syringic acid against indomethacin-induced gastric ulcer, with most of these studies primarily focusing on oxidative stress, inflammation, and apoptosis. However, the molecular mechanisms underlying the gastroprotective effects of syringic acid have not yet been fully elucidated. In particular, information regarding the role of key signaling pathways such as Nrf-2/HO-1, NF-κB/iNOS, and p38 MAPK, as well as oxidative DNA damage, in indomethacin-induced gastric injury remains limited. Therefore, the present study aimed to investigate the gastroprotective effects of syringic acid in an indomethacin-induced gastric ulcer model through a comprehensive multi-level approach, including biochemical, molecular, histopathological, immunofluorescence, and in silico analyses.

## Materıals and methods

### Animals

A total of 84 adult male Sprague–Dawley rats, 12 weeks of age and weighing approximately 220–250 g, were obtained from the Experimental Research and Application Center of Atatürk University (Erzurum, Türkiye). The rats were housed at a controlled temperature of 23 ± 2 °C with 55 ± 5% relative humidity under a 12-h light/dark cycle. They had free access to tap water and standard pellet chow (Bayramoğlu Yem ve Un Sanayi Ticaret A.Ş., Erzurum).

### Chemicals used

SA (≥ 99.76% purity, BLDpharm) was obtained for use as a gastroprotective agent. INDO (≥ 99% purity, Bostonchem) was employed to induce gastric ulcers. OMP (≥ 98% purity, BLDpharm) served as the positive control. Phosphate buffer (Sigma), adjusted to pH 7.4, was routinely used for diluting tissues during homogenization. Sevoflurane (100% liquid, Abbvie Laboratories, Istanbul, Türkiye) was administered as an anesthetic at the end of the experiment to enable the collection of gastric tissues from the rats.

### Experimental design

In this study, a gastric ulcer model was induced in rats using INDO at a dose of 100 mg/kg (Sengül and Gelen 2019) administered intragastrically (i.g.). Three different doses of SA (5, 50, and 100 mg/kg) (Sabahi et al. 2020) were given via the same route for 14 consecutive days (Ren et al. 2019). At the start of the experiment, all animals were weighed to standardize body weights and then randomly assigned to seven groups. Following group allocation, each was designated according to the experimental protocol, which was then implemented for the 14-day period as planned (Table 1).

**Table 1:** Experimental groups

Number of groups	Group names
Group 1	Control
Group 2	Indomethacin (100 mg/kg)
Group 3	Omeprazole (5 mg/kg) + Indomethacin (100 mg/kg)
Group 4	SA (5 mg/kg) + Indomethacin (100 mg/kg)
Group 5	SA (50 mg/kg) + Indomethacin (100 mg/kg)
Group 6	SA (100 mg/kg) + Indomethacin (100 mg/kg)
Group 7	SA (100 mg/kg)

Group 1 received only saline intragastrically (i.g.) for 14 days. Group 2 was given saline i.g. for 14 days, followed by a single i.g. dose of INDO on day 15. Group 3 received OMP (El-Ashmawy et al. 2016) i.g. for 14 days, and on day 15 a single i.g. dose of INDO was administered. Groups 4, 5, and 6 were given SA at doses of 5, 50, and 100 mg/kg, respectively, i.g. for 14 days, and on day 15 received a single i.g. dose of indomethacin. Group 7 was treated with SA at 100 mg/kg i.g. for 14 days only. Rats were fasted for 24 h before indomethacin administration but allowed free access to water.Twenty-four hours after INDO administration, for anesthesia, sevoflurane was administered via inhalation. After deep anesthesia was achieved, the rats were euthanized by decapitation under anesthesia to minimize pain and suffering. Gastric tissues were then quickly excised for further analyses (Underwood and Anthony 2020). From each group, gastric tissues obtained from six rats were rinsed with physiological saline and immediately fixed in formaldehyde for histopathological and immunofluorescence analyses. The gastric tissues of the remaining six rats were also rinsed with physiological saline, immediately snap-frozen in liquid nitrogen, and subsequently stored at − 80 °C until biochemical analyses were performed (Sengül and Gelen 2019).

### Preparation of gastric tissue samples

Gastric tissues, previously stored at − 80 °C according to their respective groups, were sectioned into equal portions using a scalpel. Each tissue fragment was placed into a MagNA Lyser tube containing 1.5 ml PBS (pH 7.4) and a steel bead, then sealed. The tubes were processed in a MagNA Lyser instrument (Roche) at 6000 rpm for 60 s to achieve homogenization. Following homogenization, the samples were centrifuged in a refrigerated centrifuge at 5000 rpm for 5 min. The resulting supernatants were transferred into Eppendorf tubes for biochemical analyses. Immediately after sectioning, the remaining tissue portions were returned to − 80 °C for storage.

### Biochemical analyses

At the end of the experiment, gastric tissues obtained from the rats were analyzed for the following parameters: MDA (Cat. No. E0156Ra), SOD (Cat. No. E0168Ra), GPx (Cat. No. E1172Ra), CAT (Cat. No. E0869Ra), GSH (Cat. No. EA0113Ra), TNF-α (Cat. No. E0764Ra), IL-10 (Cat. No. E0108Ra), NF-κB (Cat. No. E0287Ra), IL-1β (Cat. No. E0119Ra), iNOS (Cat. No. E0740Ra), Nrf-2 (Cat. No. E1083Ra), IL-6 (Cat. No. E0135Ra), COX-1 (Cat. No. E1245Ra), PGE2 (Cat. No. E0504Ra), p38-MAPK (Cat. No. E2473Ra), caspase-3 (Cat. No. E1648Ra), HO-1 (Cat. No. E0676Ra), and MPO (Cat. No. E0574Ra). Measurements were performed using rat-specific ELISA kits (Bioassay Technology Laboratory, BT-LAB, China) in accordance with the manufacturer’s protocols. The plates were read spectrophotometrically at 450 nm using a BioTek ELISA reader with the EPOCH II program. The results were then evaluated to determine differences between the groups.

### Macroscopic examination

The ulcer index and preventive index were calculated according to the method described by El-Ashmawy et al. The ulcer score for each group was determined as the mean ulcer count (total number of ulcers/total number of rats), with *n* = 6. The preventive index was calculated using the following formula: Preventive index = (ulcer index of the ulcer group – ulcer index of the treated group × 100)/ulcer index of the ulcer group (Küçükler et al. 2022).

### Histopathological examination

Tissue samples obtained for evaluation were fixed in 10% formaldehyde solution for 48 h, followed by routine tissue processing and embedding in paraffin blocks. Sections of 4-μm thickness were prepared from each block and stained with hematoxylin and eosin (H&E) for histopathological assessment. The stained slides were examined under a light microscope (Olympus BX 51, Japan). Based on histopathological characteristics, the sections were graded as absent (−), mild (+), moderate (+ +), or severe (+ + +). The evaluations were performed by two pathology specialists using a blinded pathology assessment method (Danışman et al. 2023).

### Double ımmunofluorescence analysis

Tissue sections prepared for immunoperoxidase examination were placed on adhesive-coated (poly-l-lysine) slides and deparaffinized. Endogenous peroxidase activity was blocked by treatment with 3% H₂O₂ solution for 10 min. The sections were then boiled in 1% antigen retrieval solution (citrate buffer, pH 6.1, 100 ×) and allowed to cool to room temperature. To prevent nonspecific background staining, a protein block was applied for 5 min. Subsequently, an 8-OHdG primary antibody (sc-66036, dilution 1:100, USA) was applied and incubated according to the manufacturer’s instructions. As the secondary label, an immunofluorescent secondary antibody (FITC, Cat No: ab6785, dilution 1:1000) was used, and the slides were kept in the dark for 45 min. Afterwards, a second primary antibody (BAX, Cat No: sc-7480, dilution 1:100, USA) was applied, followed by incubation in line with the manufacturer’s protocol. As the secondary label for this antibody, an immunofluorescent secondary antibody (Texas Red, Cat No: ab6719, dilution 1:1000, UK) was used, with slides kept in the dark for 45 min. Finally, DAPI with mounting medium (Cat. No.: D1306, dilution 1:200, UK) was applied to the sections, which were left in the dark for 5 min before being coverslipped. The stained sections were examined using a Zeiss AXIO microscope (Germany) equipped with a fluorescence attachment. Immunofluorescence (IF) staining positivity was quantitatively evaluated using the ImageJ analysis software. In the images obtained from all groups under the same microscope and camera settings, staining intensity was calculated as mean intensity within selected regions of interest (ROI). The resulting mean values were used for statistical analyses. To enable reliable relative comparisons between groups, all images were captured under standardized and fixed imaging conditions (Cicek et al. 2023).

### In silico analyses

The in silico analyses were conducted using the licensed Schrödinger Maestro 2025/1 software. The indomethacin molecule was targeted toward the cyclooxygenase-1 (COX-1) receptor (PDB ID: 2OYE), and a 20 × 20 × 20 Å grid was positioned over the interaction site of the complex. Molecular docking was performed with the Glide module, followed by calculation of the binding free energy using the MM-GBSA (Molecular Mechanics/Generalized Born Surface Area) approach. Finally, pharmacophore analyses were carried out to evaluate the binding motifs of the molecule (Bolat et al. 2025).

### Statistical analysis

Biochemical analysis results were evaluated using GraphPad Prism 8.0.2 statistics program. For statistical comparisons among more than two independent groups, one-way ANOVA followed by Tukey’s post hoc test was applied, with *p* < 0.05 considered statistically significant. For multiple comparisons, Tukey’s post hoc test was used to control the type I error rate.

First, the Shapiro–Wilk test was performed to determine whether the data followed a normal distribution. For the H&E staining results, group differences were analyzed using the non-parametric Kruskal–Wallis test, followed by Dunn’s multiple comparison test. For the parametric immunohistochemical and immunofluorescence data, one-way ANOVA was applied, followed by Tukey’s post hoc test. Statistical analyses were conducted using GraphPad Prism software, and results were considered statistically significant at *p* < 0.05.

## Results

The findings regarding the effects of syringic acid in the indomethacin-induced experimental gastric ulcer model in rats are presented in this section. In our study, seven experimental groups were established: Control, INDO, OMP + INDO, SA5 + INDO, SA50 + INDO, SA100 + INDO, and SA100. Analyses were performed on oxidative stress markers, inflammatory markers, and apoptosis markers, as well as histopathological and histoimmunofluorescence evaluations of apoptosis markers. In addition, in silico analyses were conducted to evaluate the binding interaction between indomethacin and COX-1. The obtained data were presented in the form of graphs, tables, and figures, together with statistical evaluations, to demonstrate the differences among the experimental groups.

### Macroscopic findings

Macroscopic examinations were performed for the seven groups included in our experimental protocol, and the findings are presented as follows.

**Fig. 1:**
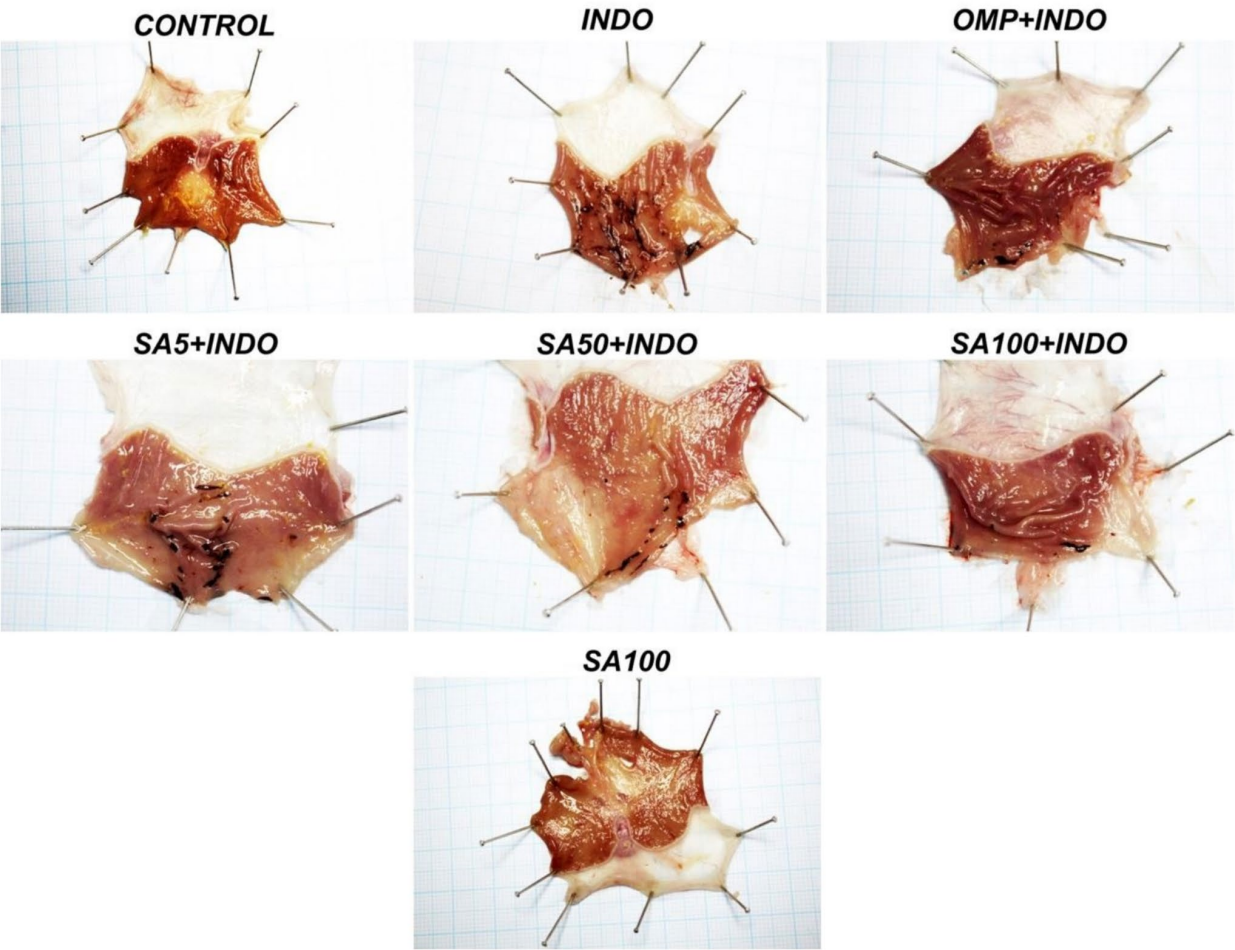
Macroscopic view of the gastric tissue (MAC)

In the macroscopic examination of the gastric tissues from the Control and SA100 groups, no pathological findings were observed. In the INDO group, severe thickening of the gastric serosa, as well as marked erosive-ulcerative lesions and hemorrhagic areas in the gastric mucosa, was detected. In the OMP + INDO group, mild erosive-ulcerative and hemorrhagic lesions were observed in the gastric mucosa, showing a statistically significant difference compared to the INDO group (*p* < 0.05). In the SA5 + INDO group, severe erosive-ulcerative and hemorrhagic lesions were present in the gastric mucosa. The SA50 + INDO group demonstrated moderate levels of erosion, ulceration, and hemorrhage, with a statistically significant difference compared to the INDO group (*p* < 0.05). In the SA100 + INDO group, mild erosion, ulceration, and hemorrhage were observed in the gastric mucosa, which also showed a statistically significant difference compared to the INDO group (*p* < 0.05) (Fig. 1). The gastric ulcer index derived from the macroscopic findings is presented in Table 2.

**Table 2:** Ulcer score, ulcer index, and preventive index of SA

	**Ulcer score**	**Ulcer index**	**Preventive index**
Control	-	-	-
INDO	26.12 ± 1.84^a^	2274	-
OMP + IND	3.72 ± 0.39^b^	145	%92.28
SA5 + INDO	24.57 ± 1.38^a^	2138	%6.3
SA50 + INDO	11.38 ± 0.92^c^	984	%74.16
SA100 + INDO	3.77 ± 0.68^b^	151	%89.13
SA100	-	-	-

The data shown for ulcer scores are expressed as mean ± SE (*n* = 6 rats/group). Different letters (a, b, c) in the columns indicate a statistically significant difference (*p* < 0.05).

### Gastric tissue oxidative stress parameters in experimental groups

In this study, oxidative stress markers, including SOD, CAT, GPx activities, and GSH levels, were evaluated. All parameters were significantly decreased in the INDO group compared to the control and protective groups (*p* < 0.0001). SA administration, particularly at doses of 50 and 100 mg, dose-dependently increased antioxidant enzyme activities and GSH levels in the ulcer-induced groups and markedly reversed the reductions caused by INDO. Although increases were observed in the SA5 + INDO group, these changes were not statistically significant (*p* > 0.05). No significant differences were observed between the control group and the OMP + INDO, SA100 + INDO, and SA100 groups (*p* > 0.05), whereas significant decreases compared to the control group persisted in the INDO, SA5 + INDO, and partially SA50 + INDO groups. These findings indicate that SA, particularly at higher doses, attenuates INDO-induced oxidative damage by enhancing the antioxidant defense system. Gastric tissue MDA levels were significantly increased in the INDO group (*p* < 0.0001). SA administration dose-dependently reduced MDA levels in the ulcer-induced groups, and this reduction was statistically significant at all doses. No significant differences were observed between the control group and the OMP + INDO, SA100 + INDO, and SA100 groups, whereas elevated MDA levels persisted in the INDO and low- and moderate-dose SA-treated groups compared to the control group. These results indicate that SA suppresses oxidative damage by reducing lipid peroxidation. Gastric tissue Nrf-2 and HO-1 levels were significantly decreased in the INDO group compared to the control and protective groups (*p* < 0.0001). SA administration, particularly at doses of 50 and 100 mg, dose-dependently increased both parameters and markedly reversed the INDO-induced suppression. Nrf-2 levels were significantly increased in the SA50 + INDO group (*p* < 0.001), whereas the increases observed in the SA5 + INDO group did not reach statistical significance for either Nrf-2 or HO-1 (*p* > 0.05). No significant differences were observed between the control group and the OMP + INDO, SA100 + INDO, and SA100 groups (*p* > 0.05), while significant decreases persisted in the INDO, SA5 + INDO, and SA50 + INDO groups compared to the control group (*p* < 0.0001). These findings indicate that SA exerts a strong protective effect against oxidative stress-induced damage, particularly at higher doses, by activating the Nrf-2/HO-1 signaling pathway (Table 3).

**Table 3:** Effects of SA against INDO-induced oxidative stress in rats (*n* = 6)

Parameters	Control	INDO	OMP + INDO	SA5 + INDO	SA50 + INDO	SA100 + INDO	SA100
SOD (ng/mL)	4.58 ± 0.67^a^	2.58 ± 0.65^b^	4.42 ± 0.31^a^	2.99 ± 0.65^b^	3.73 ± 0.59^c^	4.71 ± 0.67^a^	4.63 ± 0.51^a^
CAT (ng/mL)	58.61 ± 7.24^a^	31.72 ± 9.23^b^	55.18 ± 7.66^a^	30.01 ± 8.70^b^	44.86 ± 5.36^c^	55.42 ± 12.63^a^	55.76 ± 16.30^a^
GSH (ng/mL)	58.95 ± 6.87^a^	31.45 ± 7.55^b^	54.51 ± 9.27^a^	34.80 ± 4.85^b^	45.02 ± 6.32^c^	59.20 ± 5.38^a^	60.11 ± 10,17^a^
GPx (ng/mL)	1206.00 ± 198.81^a^	543.01 ± 320.15^b^	1166.13 ± 201.84^a^	613.37 ± 172.85^b^	798.11 ± 149.77^c^	1178.94 ± 218.71^a^	1310.44 ± 170.66^a^
MDA (ng/mL)	1.75 ± 0.17^a^	2.85 ± 0.27^b^	1.84 ± 0.41^a^	2.57 ± 0.16^c^	2.14 ± 0.24^d^	1.79 ± 0.43^ad^	1.73 ± 0.21^a^
Nrf-2 (ng/mL)	1432.43 ± 452.05^a^	526.84 ± 112.78^b^	1314.73 ± 205.26^a^	617.85 ± 166.05^b^	867.51 ± 273.56^c^	1301.55 ± 316.15^a^	1522.20 ± 267.61^a^
HO-1 (ng/mL)	7.45 ± 0.69^a^	3.58 ± 0.45^b^	7.12 ± 1.32^a^	3.69 ± 0.68^b^	4.90 ± 1.23^c^	7.04 ± 1.14^a^	7.21 ± 0.69^a^

Letters other than “a” on the same line indicate a statistically significant difference at *p* < 0.05. Values are expressed as mean ± SEM.

### Gastric tissue ınflammation parameters in the experimental groups

**Fig. 2:**
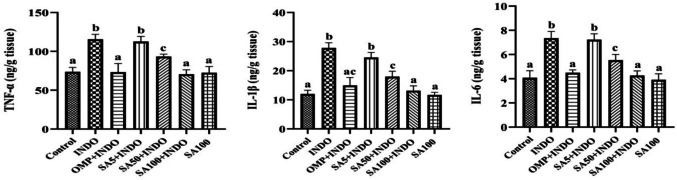
Gastric tissue TNF-α, IL-1β, and IL-6 levels in experimental groups (*n* = 6). Different letters (a, b, c) in the figure indicate statistically significant differences (*p* < 0.05). Results are expressed as mean ± SEM

As shown in Fig. 2, gastric tissue TNF-α, IL-1β, and IL-6 levels were significantly increased in the INDO group compared to the control, OMP + INDO, SA50 + INDO, SA100 + INDO, and SA100 groups (*p* < 0.0001). No significant difference was observed between the INDO and SA5 + INDO groups (*p* > 0.05), whereas TNF-α, IL-1β, and IL-6 levels were significantly decreased in the SA50 + INDO and SA100 + INDO groups compared to the INDO group, showing a dose-dependent reduction (*p* < 0.001). No significant differences were found between the control group and the OMP + INDO, SA100 + INDO, and SA100 groups (*p* > 0.05), whereas significant differences were observed between the control group and the INDO, SA5 + INDO, and SA50 + INDO groups (*p* < 0.0001–0.01). These findings indicate that syringic acid exerts a marked anti-inflammatory effect by dose-dependently reducing elevated TNF-α, IL-1β, and IL-6 levels in indomethacin-induced gastric ulcer.

**Fig. 3:**
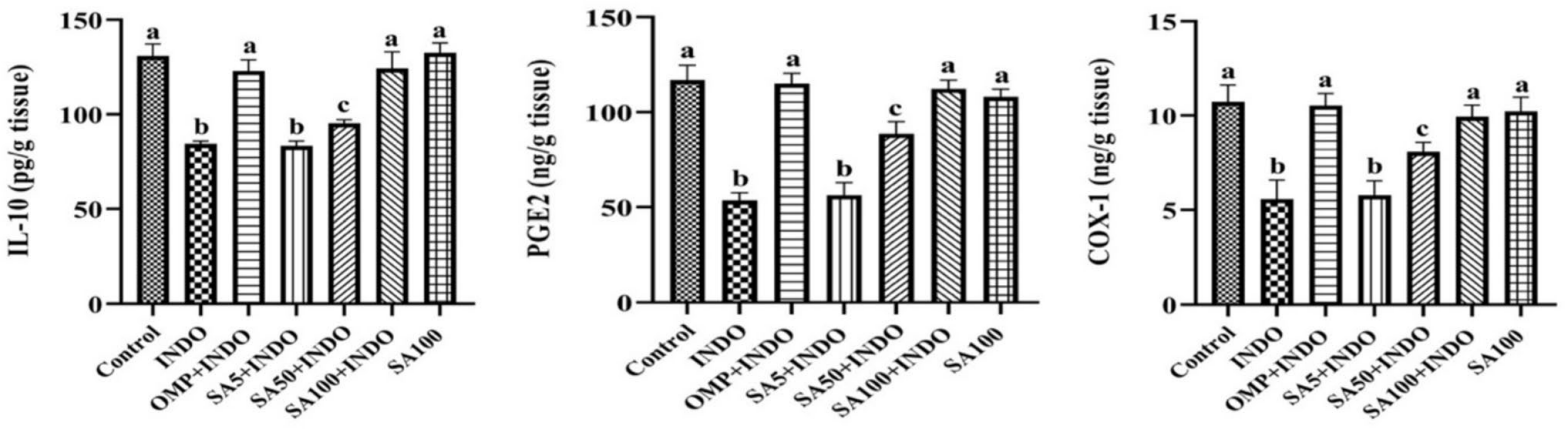
Gastric tissue IL-10 and PGE₂ levels and COX-1 activity in experimental groups (*n* = 6). Different letters (a, b, c) in the figure indicate statistically significant differences (*p* < 0.05). Results are expressed as mean ± SEM

As shown in Fig. 3, gastric tissue anti-inflammatory IL-10 levels, PGE2 levels, and COX-1 activity were significantly decreased in the INDO group compared to the control, OMP + INDO, SA50 + INDO, SA100 + INDO, and SA100 groups (*p* < 0.0001). No statistically significant difference was observed between the INDO and SA5 + INDO groups (*p* > 0.05), whereas IL-10 levels, PGE2 levels, and COX-1 activity were significantly increased in the SA50 + INDO and SA100 + INDO groups compared to the INDO group, showing a dose-dependent increase (*p* < 0.05–0.001). No significant differences were found between the control group and the OMP + INDO, SA100 + INDO, and SA100 groups (*p* > 0.05), whereas statistically significant differences were observed between the control group and the INDO, SA5 + INDO, and SA50 + INDO groups (*p* < 0.0001–0.001). These findings indicate that syringic acid dose-dependently restored decreased IL-10 and PGE2 levels and COX-1 activity in the indomethacin-induced ulcer model, thereby exerting a protective effect on gastric mucosal defense mechanisms.

**Fig. 4:**
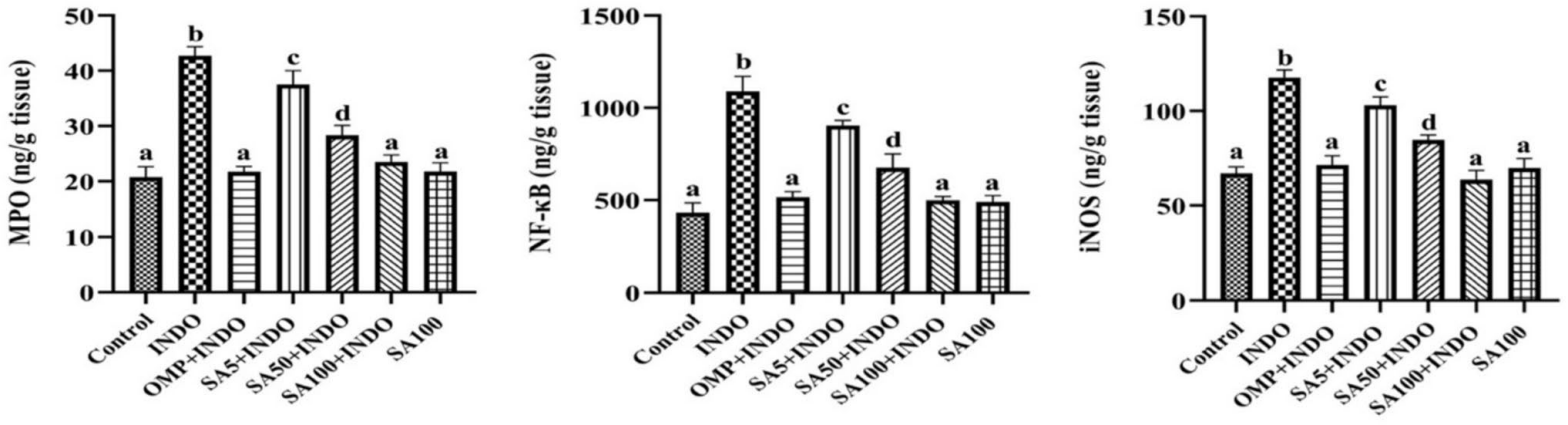
Gastric tissue NF-κB level and MPO, iNOS activities in experimental groups (*n* = 6). Different letters (a, b, c, d) in the figure indicate statistically significant differences (*p* < 0.05). Results are expressed as mean ± SEM

As shown in Fig. 4, gastric tissue MPO activity, NF-κB level, and iNOS activity were significantly increased in the INDO group compared to the control, OMP + INDO, SA50 + INDO, SA100 + INDO, and SA100 groups (*p* < 0.0001). In the SA-treated ulcer groups, MPO activity, NF-κB level, and iNOS activity were decreased in a dose-dependent manner compared to the INDO group. In the SA5 + INDO group, MPO and iNOS activities were significantly decreased compared to the INDO group (*p* < 0.001), whereas NF-κB levels were significantly decreased in all SA-treated groups compared to the INDO group (*p* < 0.0001). No significant differences were observed between the control group and the OMP + INDO, SA100 + INDO, and SA100 groups (*p* > 0.05), whereas significant differences were found between the control group and the INDO, SA5 + INDO, and SA50 + INDO groups (*p* < 0.0001). These findings indicate that syringic acid exerts a dose-dependent anti-inflammatory effect by suppressing MPO, NF-κB, and iNOS, which are associated with neutrophil infiltration and inflammatory signaling pathways, in indomethacin-induced gastric ulcer.

### Gastric tissue apoptosis parameters in the experimental groups

**Fig. 5:**
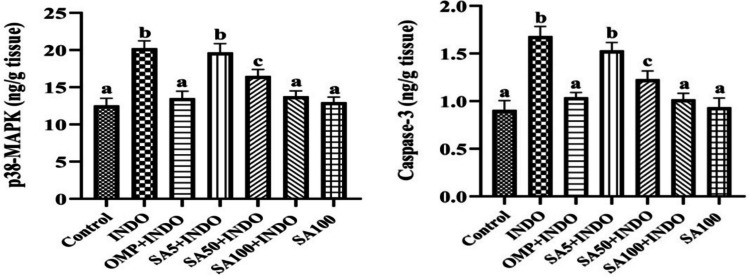
Gastric tissue p38-MAPK level and caspase-3 activity in experimental groups (*n* = 6). Different letters (a, b, c) in the figure indicate statistically significant differences (*p* < 0.05). Results are expressed as mean ± SEM

As shown in Fig. 5, gastric tissue p38-MAPK levels and caspase-3 activity were significantly increased in the INDO group compared to the control, OMP + INDO, SA50 + INDO, SA100 + INDO, and SA100 groups (*p* < 0.0001). No statistically significant difference was observed between the INDO and SA5 + INDO groups (*p* > 0.05), whereas p38-MAPK levels and caspase-3 activity were significantly decreased in the SA50 + INDO and SA100 + INDO groups compared to the INDO group, showing a dose-dependent reduction. No significant differences were found between the control group and the OMP + INDO, SA100 + INDO, and SA100 groups (*p* > 0.05), whereas significant differences were observed between the control group and the INDO, SA5 + INDO, and SA50 + INDO groups (*p* < 0.0001). These findings indicate that syringic acid exerts a dose-dependent protective effect by suppressing the p38-MAPK-mediated inflammatory signaling pathway and the caspase-3-associated apoptotic process in indomethacin-induced gastric ulcer.

### Histopathological findings

Histopathological examinations were performed for the seven groups included in our experimental protocol, and the findings are presented as follows.

**Fig. 6:**
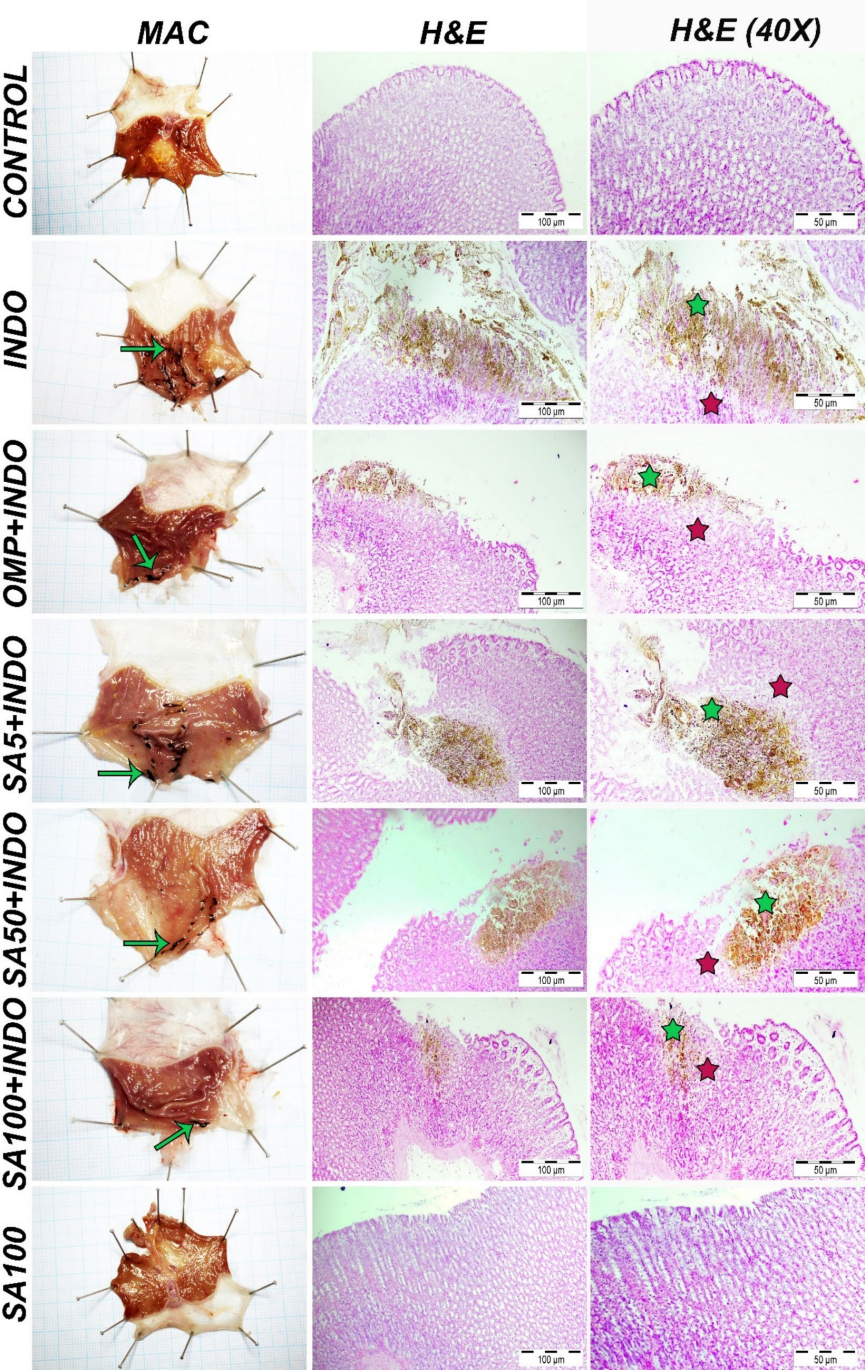
Gastric tissue, macroscopic appearance of the gastric mucosa, and histopathological findings in the gastric mucosal layer. In the MAC column, ulcerative areas macroscopically observed in the gastric mucosa are indicated by green arrows. In the H&E (40 ×) column, ulcerative areas are shown with green arrows, while necrotic areas are indicated by red arrows. Staining: H&E. Scale bar, 100 and 50 µm

In the control group, gastric tissues appeared normal upon histopathological examination. In the INDO group, histopathology of the gastric tissues revealed widespread desquamated epithelium and necrotic masses in the lumen, severe erosions and ulcerative foci extending to the lamina muscularis in the mucosal epithelium. Severe degeneration and necrosis were observed in the mucosal epithelial cells, accompanied by intense inflammation in these regions. Severe edema was detected in the submucosa, and the blood vessels showed pronounced hyperemia. In the OMP + INDO group, the mucosal layer exhibited mild erosion and mild necrotic masses, with mild degeneration, necrosis, and inflammation in the mucosal epithelial cells, and mild hyperemia in the blood vessels. In the SA5 + INDO group, severe necrotic masses and desquamated epithelial cells were observed in the gastric lumen, along with severe erosions and ulcerations in the gastric mucosa. Moderate degeneration and necrosis were present in the mucosal epithelial cells, accompanied by severe inflammation in these regions. Severe edema was observed in the submucosa, and pronounced hyperemia was detected in the blood vessels. In the SA50 + INDO group, histopathological examination of the gastric tissues revealed moderate necrotic masses, desquamated epithelial cells, and erythrocytes in the lumen, along with moderate erosive ulcers in the gastric mucosa. Moderate degeneration and mild necrosis were observed in the mucosal epithelial cells, accompanied by moderate inflammation in these areas. Mild edema was present in the submucosa, and moderate hyperemia was noted in the blood vessels. In the SA100 + INDO group, mild erosion was observed in the mucosal layer, with mild degeneration, necrosis, and inflammation in the mucosal epithelial cells, and mild hyperemia in the blood vessels. A statistically significant difference was detected compared to the INDO group (*p* < 0.05). In the SA100 group, histopathological examination of the gastric tissues revealed normal histological architecture (Fig. 6).

### Immunofluorescence staining findings

Immunofluorescence examinations were performed for the seven groups included in our experimental protocol, and the findings are presented as follows.

**Fig. 7:**
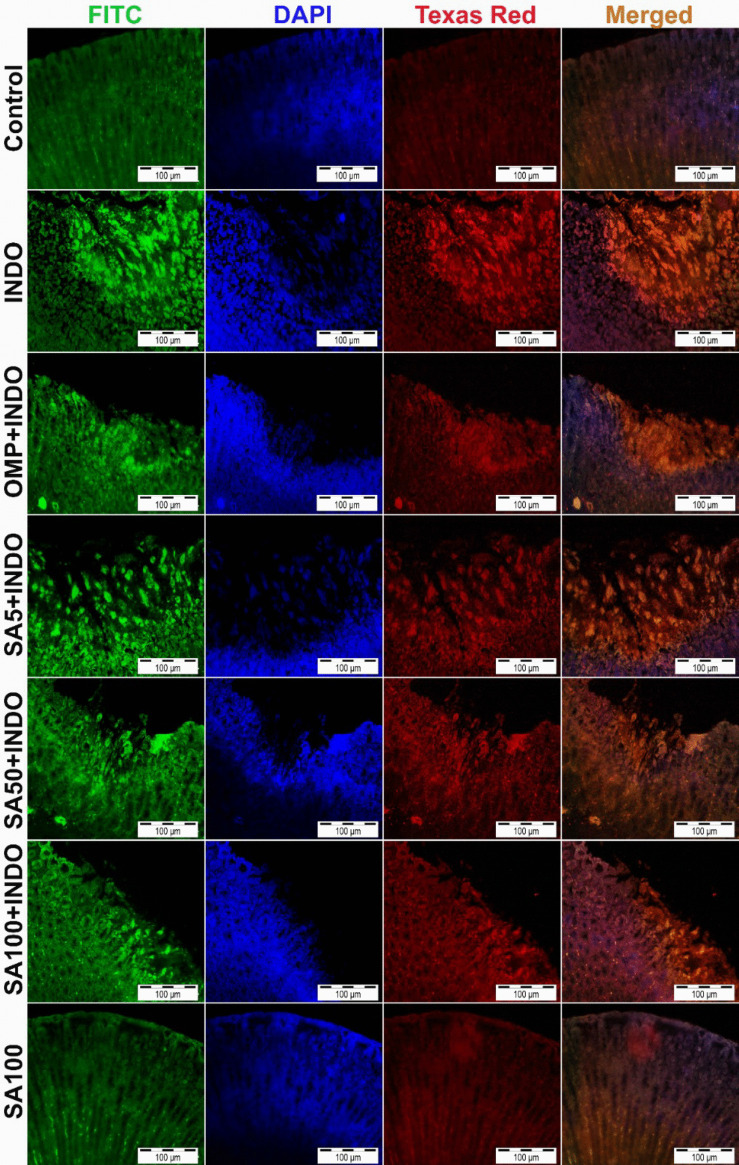
Gastric tissue, 8-OHdG expression in mucosal epithelial cells (FITC) and BAX expression (Texas Red), IF, Bar 100 µm

**Fig. 8:**
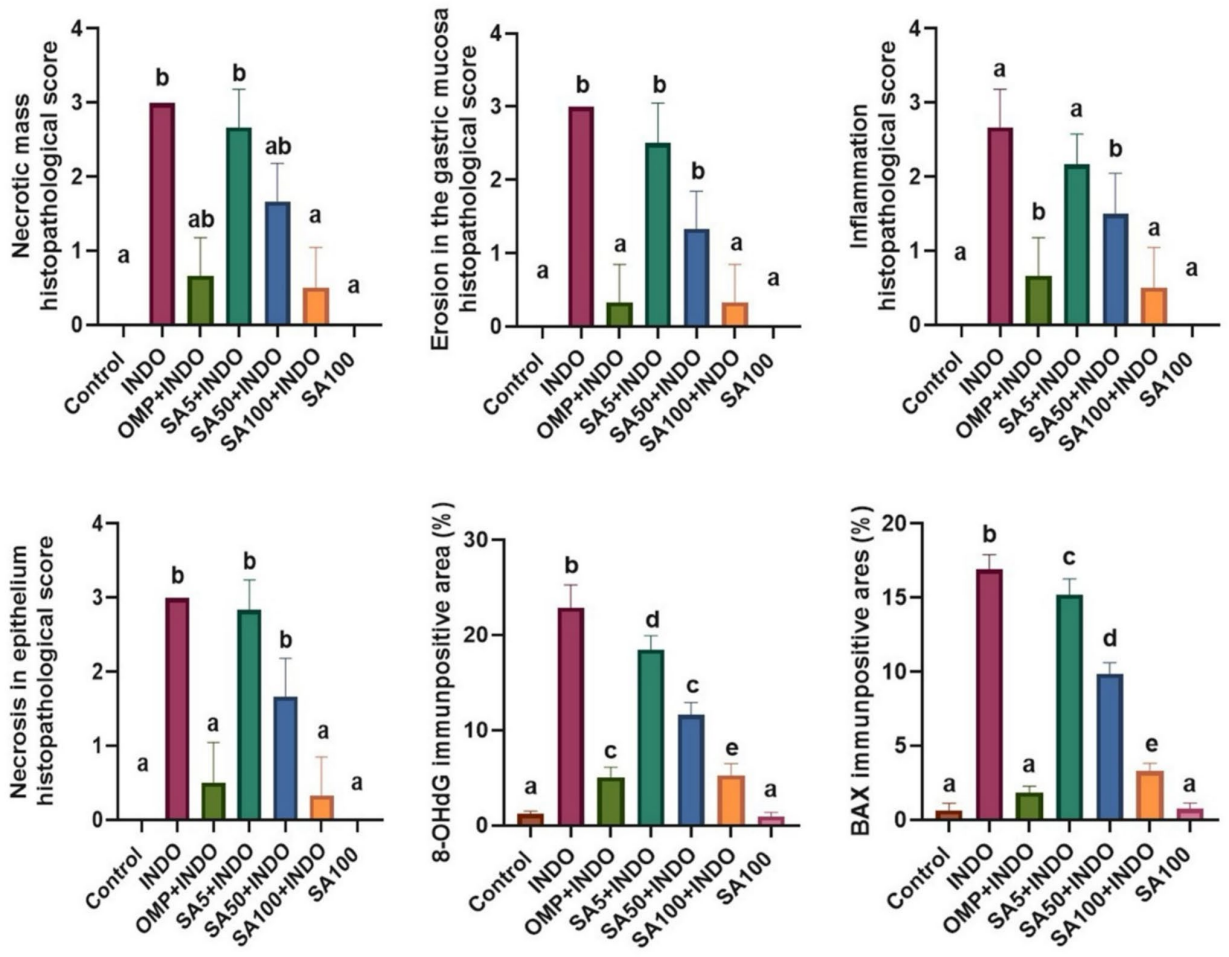
Histopathological scores determined by a blinded pathology method in gastric tissues and the statistical analysis data of these scores, together with the values of parametric immunopositive staining calculated using ImageJ in fluorescence staining and their corresponding statistical analysis data. Kruskal–Wallis and Dunn’s tests were used for the evaluation of histopathological data. One-way ANOVA followed by Tukey’s test was used for the analysis of parametric IF data. Different letters compared to control (a) indicate statistically significant differences (*p* < 0.05). Data are presented as mean ± SD

In the control group, gastric tissues examined by immunofluorescence staining showed negative 8-OHdG and BAX expression. In the INDO group, severe intracytoplasmic 8-OHdG and BAX expression was observed in mucosal epithelial cells surrounding necrotic masses in the gastric mucosal layer. In the OMP + INDO group, mild cytoplasmic 8-OHdG and BAX expression was detected in mucosal cells surrounding necrotic masses in the gastric mucosal layer. In the SA5 + INDO group, severe intracytoplasmic 8-OHdG and BAX expression was observed in mucosal epithelial cells around necrotic masses in the gastric mucosal layer. In the SA50 + INDO group, moderate intracytoplasmic 8-OHdG and BAX expression was observed in mucosal cells surrounding necrotic masses in the gastric mucosal layer. In the SA100 + INDO group, mild intracytoplasmic 8-OHdG and BAX expression was seen in mucosal epithelial cells around necrotic masses in the gastric mucosal layer. In the SA100 group, gastric tissues examined by immunofluorescence staining showed negative 8-OHdG and BAX expression (Fig. 7). Histopathological scores determined by a blinded pathology method in gastric tissues, along with the statistical analysis data of these scores, as well as the values of parametric immunopositive staining calculated using ImageJ in fluorescence staining and their corresponding statistical analysis data, are presented in Fig. 8.

### In silico analysis between INDO and COX-1

**Fig. 9:**
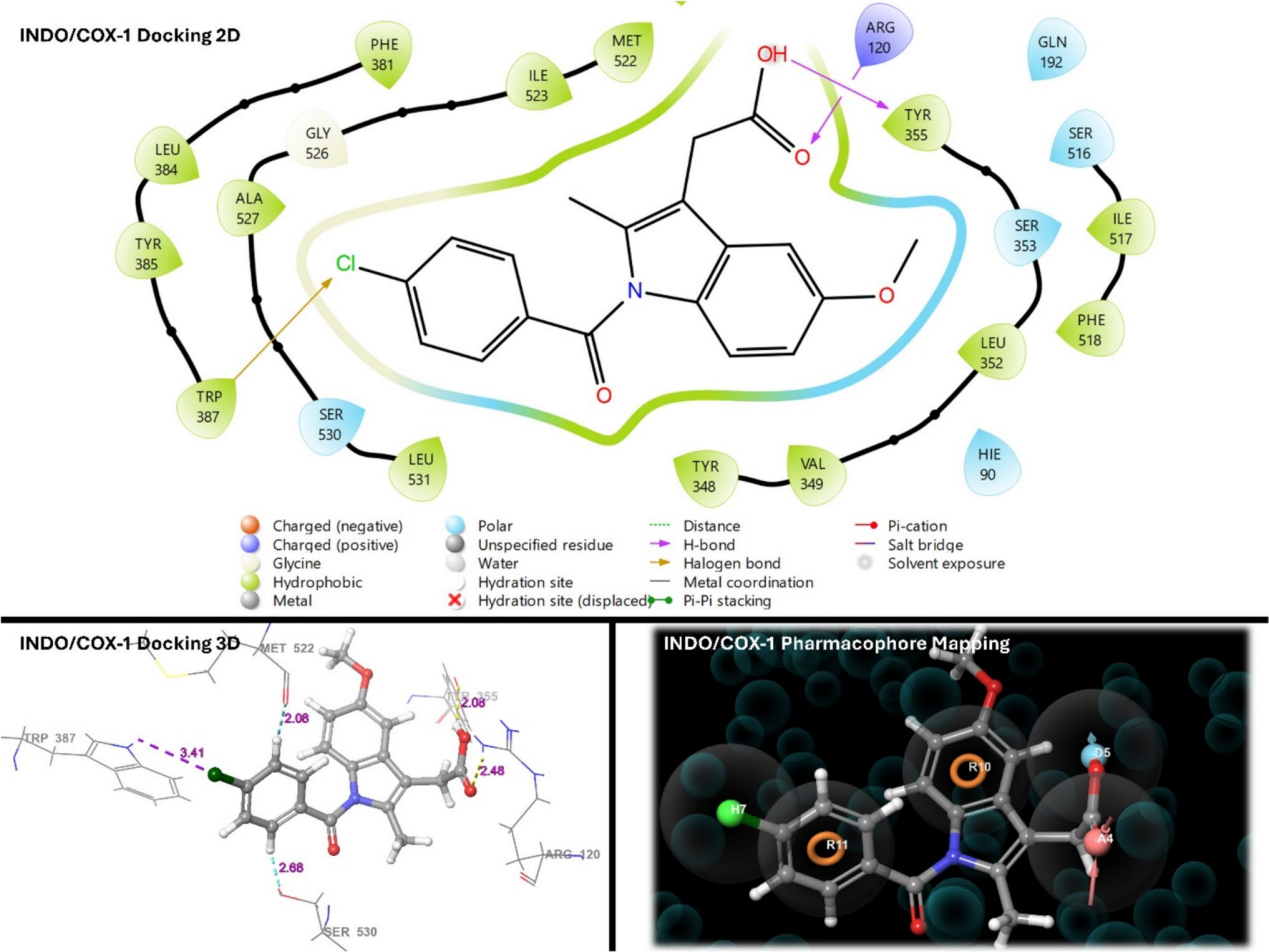
INDO/COX-1 2D, 3D, and pharmacophore mapping ımages

The docking score of indomethacin against the COX-1 receptor was − 10.1952, with a Glide energy of − 51.1923 kcal/mol. These values indicate that the drug binds to the target receptor with high affinity. In particular, docking scores below − 10 are considered indicative of pharmacologically significant binding. The notably low Glide energy suggests that the binding complex is highly stable, indicating that indomethacin has strong potential as a COX-1 inhibitor (Fig. 9, Table 4).

**Table 4:** Molecular binding analysis scores

Binding scores	INDO/COX-1
Docking scores	− 10.1952
Glide energy	− 51.1923

According to the docking analysis, indomethacin forms various hydrogen bonds, halogen bonds, and aromatic interactions with the COX-1 receptor. Specifically, it forms an aromatic hydrogen bond with SER530 at a distance of 2.68 Å, a hydrogen bond with MET522 at 2.08 Å, and a classical hydrogen bond with ARG120 at 2.48 Å. These interactions indicate that the binding is both directionally and energetically stable. Additionally, the formation of a halogen bond with TRP387 (3.41 Å) suggests that the presence of chlorine in the drug enhances its binding effect. A strong hydrogen bond is also formed with TYR355. Collectively, these bonds demonstrate that indomethacin binds to the active site of COX-1 in a highly specific manner (Table 5).

**Table 5:** Detailed table showing the distance between the amino acid to which the ligand binds and the atoms it interacts with, as determined by molecular docking analysis

Docking complex	Aminoacid	Atom1 (receptor)	Atom2 (ligand)	Distance (Angstrom = Å)
**INDO/COX-1**	SER530 (aromatic H bond)	O:3965	H:34	2.68
	TRP387 (halogen bond)	H:6975	Cl:1	3.41
	MET522 (aromatic H bond)	O:3903	H:37	2.08
	ARG120 (H bond)	H:8813	O:5	2.48
	TYR 355 (H bond)	O:2584	H:41	2.08

The total binding free energy (ΔG_bind) was calculated as − 68.41 kcal/mol. This very low value confirms the high stability of the molecule–receptor complex and the strong binding of indomethacin. Examining the individual energy contributions, van der Waals (− 46.00), lipophilic (− 28.22), and Coulombic (− 23.66) interactions were found to contribute significantly to binding. Although the binding solvent desolvation energy (Solv_GB) was positive (+ 29.44), this typically represents the energetic cost associated with binding from the solvent environment to the target and is offset by other negative energy contributions. Hydrogen bonds also contributed, with a value of − 1.34. Overall, the MM-GBSA analysis indicates that this compound possesses strong inhibitory potential against COX-1 (Table 6).

**Table 6:** MM-GBSA analysis results

MM-GBSA results	INDO/COX-1
r_psp_MMGBSA_dG_Bind	− 68.41
r_psp_MMGBSA_dG_Bind_Coulomb	− 23.66
r_psp_MMGBSA_dG_Bind_Covalent	3.57
r_psp_MMGBSA_dG_Bind_Hbond	− 1.34
r_psp_MMGBSA_dG_Bind_Lipo	− 28.22
r_psp_MMGBSA_dG_Bind_Packing	− 2.20
r_psp_MMGBSA_dG_Bind_SelfCont	0.00
r_psp_MMGBSA_dG_Bind_Solv_GB	29.44
r_psp_MMGBSA_dG_Bind_Solv_SA	(blank)
r_psp_MMGBSA_dG_Bind_vdW	− 46.00

Pharmacophore matching analysis reveals the key binding motifs used by INDO when interacting with the COX-1 receptor. The D5 feature, which scored highest at − 1.47, indicates a region capable of acting as a hydrogen bond donor or acceptor. The R10 and R11 features represent hydrophobic interactions with aromatic rings, while regions such as H7 (hydrophobic area) and A4 (hydrogen bonding) are critical points supporting drug–receptor interactions. The three-dimensional pharmacophore modeling of these features demonstrates that the molecule binds to the target with high specificity and that this interaction is supported by various pharmacophoric characteristics (Table 7).

**Table 7:** Pharmacophore mapping analysis

Rank	Feature_label	Score	*X*	*Y*	*Z*	Type	Num	Source
1	D5	− 1.47	253.0103	111.9916	− 39.9007	D	4	HBond
2	R10	− 1.44	252.4483	108.0851	− 41.0923	R	9	RingChemscoreHphobe
3	R11	− 1.35	248.7389	104.9046	− 39.2912	R	10	RingChemscoreHphobe
4	H7	− 0.3	245.99	103.8699	− 40.4058	H	6	PhobEn
5	A4	− 0.16	251.8681	111.856	− 37.0183	A	3	HBond

## Dıscussıon

A gastric ulcer, defined as a lesion characterized by disruption of the gastric tissue integrity, may arise from several etiological factors, among which oxidative stress plays a significant role. Excessive generation of reactive oxygen species beyond antioxidant defense capacity leads to oxidative stress, resulting in lipid peroxidation, protein and DNA damage, and subsequent epithelial disruption and cell death in the gastric mucosa, thereby promoting ulcer development (Kahraman et al. 2003). The initial stage of cellular injury is characterized by peroxidation of membrane lipids, which represents a key event in oxidative stress-mediated gastric damage (Kwiecien et al. 2012). Within the gastric mucosa, homeostasis is maintained by the balance between ROS production and antioxidant enzymes. When this equilibrium is disrupted, oxidative stress may induce excessive ROS accumulation and malondialdehyde formation. MDA, the primary end-product of lipid peroxidation, directly reflects mucosal injury and mediates the progression of gastric lesions. Hence, MDA is regarded as a critical biomarker of ROS-induced peroxidation, providing key evidence for the involvement of oxidative stress in gastric ulcer pathogenesis (Yuksel et al. 2012). Nonsteroidal anti-inflammatory drugs (NSAIDs) are known to contribute to the development of pathological mechanisms in gastric tissue by inducing oxidative stress through enhanced lipid peroxidation, protein oxidation, and increased production of reactive oxygen species (ROS) (Naito and Yoshikawa 2006). Indomethacin, in particular, has been reported to increase MDA levels in gastric tissue, reflecting elevated lipid peroxidation and oxidative damage (Kwiecien et al. 2014). In an experimental ulcer model, indomethacin administration significantly increased MDA levels in gastric tissue, whereas antioxidant treatment at different doses reduced MDA concentrations and provided partial or complete protection of the gastric mucosa, indicating its protective potential against INDO-induced oxidative damage (Boyacioğlu et al. 2016). Furthermore, in a rat study investigating the neuroprotective effects of syringic acid, SA treatment was shown to reduce MDA levels following cerebral ischemia (Tokmak et al. 2015). Consistent with the literature, the present study demonstrated that MDA levels were significantly higher in the INDO group compared with the control and treatment groups in the indomethacin-induced gastric ulcer model. Moreover, SA administration decreased MDA levels in a dose-dependent manner in ulcer-induced groups, suggesting that SA effectively prevents lipid peroxidation and attenuates oxidative damage in gastric tissue.

The antioxidant defense system is an important mechanism that protects tissues against oxidative damage caused by reactive oxygen species (Reuter et al. 2010). Antioxidant enzymes such as SOD, CAT, and GPx, along with GSH, play a critical role in reducing oxidative stress (Ighodaro and Akinloye 2018). Previous studies have reported that nonsteroidal anti-inflammatory drugs, particularly indomethacin, induce oxidative stress, leading to a decrease in antioxidant enzyme activities and GSH levels, which contributes to increased gastric mucosal damage (Suleyman et al. 2010). Indeed, experimental ulcer models induced by indomethacin have demonstrated a significant reduction in SOD, CAT, and GPx activities as well as GSH levels (Odabasoglu et al. 2006; Kwiecien et al. 2012). In contrast, syringic acid has been reported to exert a protective effect against oxidative damage by increasing antioxidant enzyme activities and GSH levels in different experimental models (Shahzad et al. 2019; Tokmak et al. 2015). Consistent with the literature, the findings of the present study showed that SOD, CAT, and GPx activities and GSH levels were significantly decreased in the INDO group. However, in the SA-treated ulcer groups, these antioxidant parameters increased in a dose-dependent manner, and this decrease was markedly reversed, particularly at higher doses. These findings suggest that syringic acid exerts a protective effect against indomethacin-induced oxidative damage by enhancing the antioxidant defense system.

The Nrf-2/HO-1 signaling pathway plays a critical role in protecting cells from oxidative stress-induced damage by restoring endogenous antioxidant enzymes. Nrf-2 is an important transcription factor involved in cellular defense against oxidative damage. Under normal conditions, this factor binds to its cytoplasmic negative regulator, Kelch-like ECH-associated protein 1 (Keap1), remaining inactive. Upon increased levels of ROS, Nrf-2 becomes activated and dissociates from Keap1. The activated Nrf-2 translocates to the nucleus, where it binds to Maf proteins to stimulate antioxidant response elements and activate antioxidant enzymes such as HO-1, SOD, CAT, and GPx (Kensler et al. 2007). By enhancing the cell’s antioxidant capacity, Nrf-2 plays a key role in protecting against the harmful effects of ROS. Moreover, Nrf-2 exerts protective effects in a variety of diseases associated with inflammation, oxidative stress, toxic agents, and other stress factors, including cancer, neurodegenerative and cardiovascular diseases, and diabetes. Functioning as a sensor of intracellular oxidative stress, Nrf-2 activity increases in response to elevated ROS levels, thereby enhancing cellular antioxidant defenses and mitigating ROS-induced damage (Chowdhury et al. 2022). HO-1 is an enzyme present in cells that plays a critical role in protecting them against oxidative stress, inflammation, and other stressors. It catalyzes the degradation of heme-containing proteins, leading to the release of heme groups. Free heme can cause cellular damage under conditions of oxidative stress and inflammation. HO-1 mitigates this toxicity by converting free heme into biliverdin and Fe2⁺ through reactions with oxygen and carbon monoxide. In this way, it prevents the harmful effects of heme and enhances the cell’s antioxidant capacity. HO-1 expression increases in cells exposed to oxidative stress, inflammation, cancer, ischemia, heavy metals, hypoxia, and other stressors. It also modulates signaling pathways to regulate apoptosis and inflammatory responses. As a stress-inducible enzyme, HO-1 produces equimolar amounts of biliverdin, ferrous iron, and carbon monoxide, acting as a reliable antioxidant and cytoprotective agent. Upregulation of HO-1 leads to increased accumulation of iron, bilirubin, and carbon monoxide, which reduces the susceptibility of cells to oxidative damage. Among enzymes capable of protecting against oxidative stress, HO-1 is particularly notable for its cytoprotective effects, as it not only neutralizes reactive oxygen species but also provides protection against apoptosis (Ryter et al. 2006). Drugs and other therapeutic interventions that enhance HO-1 activity may have potential benefits in the treatment of various diseases associated with oxidative stress and inflammation (Albarakati et al. 2020). Several studies have reported that INDO-induced ulceration is associated with decreased expression of Nrf-2 and HO-1, indicating a potential contribution of INDO-induced oxidative stress to mucosal damage (Balaha et al. 2022). Recent studies have demonstrated that the protective effects of syringic acid are closely associated with the activation of the Nrf2/HO-1 signaling pathway (Zhao et al. 2025). Syringic acid has been reported to activate the PI3K/Akt-mediated Nrf2/HO-1 pathway and attenuate oxidative stress and inflammation in doxorubicin-induced hepatotoxicity (Alwaili et al. 2025). Similarly, in cisplatin-induced toxicity and experimental colitis models, syringic acid enhanced antioxidant defense by activating the Nrf2 pathway and reduced tissue damage (Demir et al. 2024; Ekhtiar et al. 2023). Consistent with these findings, the results of the present study suggest that the protective effect of syringic acid against indomethacin-induced gastric injury may be associated with activation of the Nrf2/HO-1 signaling pathway and enhancement of the antioxidant defense system.

Inflammation is a biochemical defense mechanism that protects against cellular damage. Reactive oxygen species released by immune cells at sites of inflammation are a major source of cellular injury. Cytokines play a central role in initiating and regulating immune responses. The cytokine network is a complex biological system that exerts direct or indirect effects on numerous immune and non-immune cells, and disruption of this network is known to lead to various immune system disorders (Dinarello 2000). Proinflammatory cytokines are among the major inflammatory mediators that play a significant role in the pathogenesis of gastric ulcers. Cytokines such as TNF-α, IL-1β, and IL-6 are involved in the initiation and progression of the inflammatory response and contribute to ulcer formation by promoting neutrophil infiltration, free radical production, and increased tissue damage. Indomethacin has been reported to increase inflammation in the gastric mucosa, leading to elevated levels of these proinflammatory cytokines, which play a crucial role in the aggravation of mucosal injury. Recent studies have also demonstrated that TNF-α, IL-1β, and IL-6 levels are significantly increased in gastric tissue in experimental indomethacin-induced ulcer models (Jia et al. 2023a, b; Balaha et al. 2022). Recent studies have reported that the anti-inflammatory effects of syringic acid are largely associated with the suppression of the NF-κB-mediated inflammatory signaling pathway and the subsequent reduction of proinflammatory cytokines such as TNF-α, IL-1β, and IL-6. Syringic acid has been shown to reduce TNF-α, IL-1β, and IL-6 levels and increase anti-inflammatory cytokines such as IL-10 in different experimental inflammation models, thereby regulating the inflammatory response (Fang et al. 2019; Alwaili et al. 2025). Moreover, the anti-inflammatory effects of syringic acid have been associated with the inhibition of inflammatory pathways such as NF-κB, iNOS, and COX-2, through which it suppresses the production of proinflammatory cytokines (Zhao et al. 2025). Consistent with the literature, the findings of the present study showed that TNF-α, IL-1β, and IL-6 levels were significantly increased in the INDO group compared with the control and treatment groups. However, in the ulcer groups treated with syringic acid, particularly at doses of 50 and 100 mg, the levels of these proinflammatory cytokines decreased in a dose-dependent manner. The low dose of syringic acid did not produce a significant effect on cytokine levels. These findings suggest that syringic acid reduces indomethacin-induced gastric inflammation by suppressing proinflammatory cytokine production and that its anti-inflammatory effect may be associated with inhibition of NF-κB-related inflammatory signaling pathways.

Anti-inflammatory cytokines play an important role in the regulation of the inflammatory response, and among them, IL-10 is considered one of the major regulatory cytokines responsible for suppressing inflammation and limiting tissue damage. IL-10 controls the inflammatory response and reduces tissue injury by inhibiting the production of proinflammatory cytokines (Saraiva and O’Garra 2010). In experimental indomethacin-induced gastric ulcer models, IL-10 levels have been reported to decrease, and this reduction has been associated with the exacerbation of inflammation (Jia et al. 2023a, b). Moreover, syringic acid has been reported to exert anti-inflammatory effects in different experimental models by suppressing proinflammatory mediators such as NF-κB, TNF-α, and IL-1β while increasing IL-10 levels (Ren et al. 2019). Consistent with the literature, the findings of the present study showed that IL-10 levels were significantly decreased in the INDO group, whereas IL-10 levels increased in a dose-dependent manner, particularly at higher doses, in the syringic acid–treated groups. These findings suggest that the anti-inflammatory effect of syringic acid may be associated not only with the suppression of proinflammatory cytokines but also with the upregulation of anti-inflammatory cytokines.

Neutrophil infiltration is one of the key mechanisms involved in the pathogenesis of NSAID-induced gastric injury, and this process is regulated by cytokines, chemokines, and adhesion molecules (Tarnawski and Jones 2003). Myeloperoxidase (MPO), which is predominantly found in neutrophils, is considered a specific marker of neutrophil infiltration, and increased MPO activity during inflammation is directly associated with the severity of tissue inflammation. MPO contributes to oxidative stress and subsequent tissue injury by promoting the production of reactive oxygen and reactive nitrogen species (Chen et al. 2020). Experimental studies have reported that indomethacin administration increases MPO activity and that increased neutrophil infiltration plays a significant role in the progression of gastric mucosal injury (Pineda-Pena et al. 2018). In contrast, flavonoids and phenolic compounds have been reported to exert anti-inflammatory effects by reducing MPO activity (Sinha et al. 2015). Furthermore, syringic acid has been shown to decrease MPO activity in experimental inflammation models (Fang et al. 2019). Consistent with the literature, the findings of the present study demonstrated that MPO activity was significantly increased in the INDO group, whereas MPO activity decreased in a dose-dependent manner in the syringic acid–treated groups. These findings suggest that syringic acid exerts anti-inflammatory effects against indomethacin-induced gastric injury by reducing neutrophil infiltration and associated oxidative stress.

The maintenance of gastric mucosal integrity depends on the balance between aggressive and defensive factors. The mucus–bicarbonate barrier, prostaglandins, nitric oxide, mucosal blood flow, and antioxidant systems are among the main defensive factors, whereas acid, leukotrienes, and reactive oxygen species are the major aggressive factors. Among these defensive factors, prostaglandin E2 (PGE2) is an important mediator that plays a critical role in the protection of the gastric mucosa. PGE2 enhances gastric mucosal blood flow, inhibits gastric acid secretion, promotes epithelial cell regeneration, and accelerates mucosal healing (Laine et al. 2008; Jia et al. 2023a, b). The synthesis of PGE2 occurs in two steps: arachidonic acid is first converted to prostaglandin H₂ by cyclooxygenase enzymes (COX-1 and COX-2), and prostaglandin H₂ is subsequently isomerized to PGE2 by prostaglandin E synthase. A decrease in PGE2 levels in the gastric mucosa may lead to the development of gastric ulcers and exacerbate existing ulcers. COX-1 is constitutively expressed in the gastrointestinal tract and is responsible for the synthesis of prostaglandins involved in maintaining mucosal integrity (Laine et al. 2008; Kloska et al. 2020). Nonsteroidal anti-inflammatory drugs, particularly indomethacin, non-selectively inhibit COX-1 and COX-2 enzymes, thereby reducing PGE2 synthesis and leading to gastric mucosal damage and ulcer formation (Halter et al. 2001). In line with these findings, the present study showed that PGE2 levels and COX-1 activity were significantly reduced in the INDO group. However, treatment with syringic acid, particularly at doses of 50 and 100 mg, significantly increased PGE2 levels and COX-1 activity in a dose-dependent manner. These results indicate that the gastroprotective effect of syringic acid may be associated with the restoration of the COX-1/PGE2 pathway and enhancement of gastric mucosal defense mechanisms.

Nitric oxide (NO) is an important mediator involved in the regulation of gastric mucosal blood flow, mucus secretion, and the maintenance of epithelial integrity; however, depending on its source, NO may exert both protective and cytotoxic effects. While NO derived from constitutive NOS (eNOS and nNOS) plays a protective role in maintaining mucosal integrity, excessive NO produced by inducible NOS (iNOS) reacts with superoxide radicals to form peroxynitrite, a potent oxidant that contributes to oxidative damage, inflammation, and cell death in the gastric mucosa (Kumar and Chanana 2017). Recent studies have demonstrated that NSAID-induced gastric injury is associated with increased iNOS expression, which contributes to enhanced oxidative stress and inflammation (Jia et al. 2023a, b). NF-κB is a key transcription factor that plays a central role in the regulation of inflammatory responses and controls the expression of many proinflammatory genes, including TNF-α, IL-1β, IL-6, iNOS, and COX-2. It has been reported that NF-κB activation is increased in indomethacin-induced gastric injury, leading to enhanced synthesis of proinflammatory cytokines (Carrasco-Pozo et al. 2016). Recent studies have further shown that NSAID-induced gastric mucosal injury is closely associated with activation of the NF-κB/iNOS-mediated inflammatory signaling pathway, and inhibition of this pathway has been shown to attenuate gastric injury (Zhang et al. 2022; Wang et al. 2023). Moreover, syringic acid has been reported to exert anti-inflammatory effects by suppressing NF-κB signaling and reducing the expression of inflammatory mediators and iNOS (Shaik et al. 2020). Consistent with the literature, the findings of the present study demonstrated that iNOS activity and NF-κB levels were significantly increased in the INDO group. In contrast, both iNOS activity and NF-κB levels decreased in a dose-dependent manner in the syringic acid–treated ulcer groups. These findings suggest that syringic acid exerts a protective effect against indomethacin-induced gastric injury by suppressing NF-κB-mediated inflammatory signaling and reducing iNOS expression, thereby preventing nitric oxide–mediated oxidative damage and inflammation.

Apoptosis, also known as programmed cell death, is a physiological process that eliminates cells that are no longer needed, have completed their biological function, or have been damaged, through genetically regulated mechanisms. The regulation of apoptosis involves various proteins and molecules. The Bcl-2 gene belongs to the anti-apoptotic family, whereas Bax is a member of the pro-apoptotic family. Activation of apoptotic signaling molecules suppresses Bcl-2 expression while increasing Bax expression; consequently, the activation of apoptosis signaling molecules has been reported to elevate caspase-3 gene expression (Dagdeviren 2021). The Bax protein has been reported to translocate to the outer mitochondrial membrane in response to oxidative stimuli, increasing membrane permeability and triggering cytochrome c release, thereby promoting apoptosis (Crompton 2000). In rats with indomethacin-induced gastric ulcers, Bax protein levels were found to be elevated (Liu et al. 2015). In a study investigating the protective effect of SA against apoptosis, SA was shown to downregulate Bax expression (Song et al. 2016). In the present study, Bax protein expression was evaluated by immunofluorescence, and Bax expression was found to be significantly increased in the INDO and SA5 + INDO groups compared with the control and OMP + INDO groups. In contrast, Bax expression was markedly decreased in the SA50 + INDO and SA100 + INDO groups. These findings suggest that syringic acid exerts a regulatory effect on the mitochondrial apoptotic pathway in indomethacin-induced gastric injury by suppressing Bax-mediated apoptosis, thereby providing a protective effect against gastric mucosal damage through its anti-apoptotic activity.

Caspases are key markers of apoptosis and are synthesized as inactive proenzymes that become activated in response to intrinsic or extrinsic stimuli. Among these, caspase-3 is considered one of the most important effector caspases and is regarded as a hallmark indicator of apoptosis (Li and Yuan 2008). In a study using an indomethacin-induced gastric ulcer model, caspase-3 activity was found to be increased in the INDO group (Balaha et al. 2022). Consistent with the literature, the findings of the present study demonstrated that caspase-3 levels were significantly increased in the INDO group compared with the control and treatment groups. Although a decrease in caspase-3 levels was observed in the SA5 + INDO group compared with the INDO group, this reduction was not statistically significant. In contrast, caspase-3 levels were reduced in a dose-dependent manner in the ulcer groups treated with 50 mg and 100 mg syringic acid compared with the INDO group. These findings suggest that syringic acid exerts an anti-apoptotic effect against indomethacin-induced gastric mucosal injury by suppressing caspase-3-mediated apoptotic pathways.

Mitogen-activated protein kinases are key cellular signaling molecules that transmit various extracellular signals into intracellular responses through sequential phosphorylation cascades. Several parallel subgroups have been identified, including c-Jun N-terminal kinase (JNK)**,** extracellular signal-regulated kinases (ERKs), and p38-MAPK (Bak et al. 2016). MAPKs play crucial roles in numerous biological processes such as cell proliferation, differentiation, apoptosis, stress response, migration, and survival. Gastric ulcer, accompanied by oxidative stress, can disrupt the MAPK signaling pathway, which regulates many physiological processes (Yadav et al. 2012). It is known that p38-MAPK is activated and phosphorylated in response to oxidative stress. Tissue injury and other external stimuli can induce the secretion of multiple proinflammatory cytokines, including TNF-α, IL-1β, and IL-6, subsequently triggering p38 activation (Chaparro-Huerta et al. 2008). In an indomethacin-induced GU model, the increased activity of p38-MAPK confirmed its role in gastric inflammation (Sallam et al. 2021). Consistent with the literature, the findings of the present study demonstrated that p38 MAPK levels were significantly increased in the INDO group compared with the control and treatment groups. No statistically significant difference was observed between the INDO and SA5 + INDO groups. However, p38 MAPK levels were reduced in a dose-dependent manner in the ulcer groups treated with 50 mg and 100 mg syringic acid compared with the INDO group. These findings suggest that syringic acid exerts a protective effect against indomethacin-induced gastric mucosal injury by suppressing p38 MAPK-mediated inflammatory and apoptotic signaling pathways.

Oxidative stress is one of the major mechanisms involved in gastric mucosal injury through the formation of reactive oxygen species-induced damage to DNA bases. 8-Hydroxy-2′-deoxyguanosine (8-OHdG), one of the most reliable biomarkers of oxidative DNA damage, is formed as a result of oxidative modification of the guanine base and reflects the degree of oxidative DNA damage (Valavanidis et al. 2009). It has been reported that 8-OHdG levels are an important indicator in the evaluation of NSAID-induced gastric damage and are significantly increased in indomethacin-induced gastric ulcer models (Yanaka et al. 2007; Bicer et al. 2016). Consistent with the literature, the findings of the present study demonstrated that 8-OHdG expression was significantly increased in the INDO and SA5 + INDO groups, whereas it was markedly decreased in the SA50 + INDO and SA100 + INDO groups. These findings suggest that syringic acid exerts a protective effect against indomethacin-induced gastric mucosal injury by reducing oxidative DNA damage.

It is well established that severe pathological alterations occur in the histological structure of gastric tissue in indomethacin-induced gastric ulcer models. Previous studies have reported prominent histopathological findings in indomethacin-treated groups, including mucosal edema, hemorrhage, necrosis, neutrophil infiltration, and epithelial cell loss (Jafari et al. 2022). Similarly, in a toxicity study, numerous necrotic and edematous foci were detected in the tissues of the toxic group, whereas these lesions were reduced in the groups treated with syringic acid compared to the toxic group (Okkay et al. 2022). These histopathological alterations indicate that mechanisms such as oxidative stress, inflammation, and apoptosis play important roles in the pathogenesis of gastric mucosal injury. Consistent with the literature, the histopathological findings of the present study revealed severe epithelial debris, necrotic masses within the lumen, erosions extending to the lamina muscularis, and multiple ulcerative lesions in the gastric mucosa in the INDO-treated groups. In addition, marked degeneration and necrosis of mucosal epithelial cells, severe inflammation, submucosal edema, and pronounced vascular hyperemia were observed. In contrast, histopathological damage was reduced in a dose-dependent manner in the syringic acid–treated groups. While low-dose syringic acid did not show a significant effect on histopathological findings, the 50-mg dose moderately reduced the lesions, and the 100-mg dose markedly improved the indomethacin-induced histopathological alterations. These histopathological findings are consistent with the biochemical and molecular results and indicate that syringic acid reduces gastric mucosal damage and preserves mucosal integrity through its antioxidant, anti-inflammatory, and anti-apoptotic effects.

Omeprazole, a proton pump inhibitor, exerts its anti-ulcer effect primarily by suppressing gastric acid secretion (Kangwan et al. 2014), whereas syringic acid appears to exert its protective effects through antioxidant, anti-inflammatory, and anti-apoptotic mechanisms. In the present study, although omeprazole showed significant protective effects, syringic acid, particularly at higher doses, demonstrated comparable protective effects in several biochemical and histopathological parameters. These findings suggest that syringic acid may not only act through acid suppression but may also protect gastric tissue by modulating oxidative stress, inflammation, and apoptosis-related signaling pathways. Therefore, syringic acid may have potential as a complementary therapeutic agent rather than a direct alternative to standard anti-ulcer drugs.

In the SA100 group, no statistically significant differences were observed in any of the evaluated biochemical, histopathological, or immunofluorescence parameters compared to the control group. This finding indicates that syringic acid, when administered alone, did not produce any detectable adverse or toxic effects on gastric tissue under the experimental conditions. In experimental ulcer models, the inclusion of a compound-alone group is important for evaluating the possible effects of the tested compound on tissue integrity and basal physiological processes. The findings of the present study indicate that syringic acid at the administered dose did not adversely affect gastric mucosal integrity and did not induce unfavorable changes in oxidative stress, inflammation, or apoptosis-related parameters. These findings suggest that the gastroprotective effects of syringic acid are primarily associated with its protective activity against induced injury and that it does not exert harmful effects on gastric tissue under normal physiological conditions.

Indomethacin, a non-selective cyclooxygenase (COX) inhibitor, has been associated with gastrointestinal side effects due to its high affinity for the COX-1 enzyme. COX-1 plays a protective role in the gastric tissue by mediating prostaglandin production within the gastric mucosa. Therefore, inhibition of COX-1 can weaken the gastric mucosal barrier and contribute to ulcer formation. A previous study demonstrated that indomethacin exhibits a higher binding affinity for COX-1 compared to COX-2 (Roberts et al. 2024). Through in silico analyses, the binding properties of indomethacin to the COX-1 receptor were examined in detail. The molecular docking analysis yielded a docking score of − 10.1952 and a Glide energy of − 51.1923 kcal/mol, indicating a high binding affinity of indomethacin for COX-1. In addition, the MM-GBSA analysis produced a binding free energy of − 68.41 kcal/mol, supporting the stability of the complex. Pharmacophore matching analyses revealed specific binding motifs of indomethacin within the active site of COX-1. These findings suggest that indomethacin may contribute to ulcer formation by inhibiting COX-1, thereby reducing prostaglandin production in the gastric mucosa.

The present study differs from previous studies in several important aspects. Unlike earlier reports, this study investigated the dose-dependent effects of syringic acid and evaluated its protective effects through a comprehensive analysis of multiple pathways involved in gastric ulcer pathogenesis, including oxidative stress, inflammation, apoptosis, DNA damage, and gastric mucosal defense mechanisms. In particular, the roles of the Nrf-2/HO-1 antioxidant pathway, NF-κB/iNOS inflammatory pathway, and p38 MAPK signaling pathway were investigated together for the first time in an indomethacin-induced gastric ulcer model treated with syringic acid. In addition, oxidative DNA damage was evaluated by assessing 8-OHdG expression, and molecular docking analyses were performed to investigate the interaction between indomethacin and COX-1. These findings provide a more comprehensive understanding of the molecular mechanisms underlying the gastroprotective effects of syringic acid.

One limitation of the present study is that apoptosis was evaluated based on Bax expression and caspase-3 levels, while TUNEL analysis, which directly detects apoptotic DNA fragmentation, was not performed. Therefore, future studies including TUNEL analysis are warranted to further confirm apoptotic cell death in a more comprehensive manner. Another limitation of this study is that molecular docking analysis was performed only for indomethacin and COX-1. Docking analyses investigating the interaction of syringic acid with potential molecular targets such as COX-1, COX-2, or proteins involved in the Nrf2 signaling pathway could provide further mechanistic insight and should be addressed in future studies.

## Conclusion

In conclusion, the present study demonstrated that indomethacin induces gastric ulceration through multiple interconnected pathophysiological mechanisms, including oxidative stress, inflammation, apoptosis, and impairment of the gastric mucosal defense system. Indomethacin administration increased lipid peroxidation and oxidative DNA damage while suppressing antioxidant defense mechanisms, as evidenced by decreased SOD, CAT, and GPx activities and reduced GSH levels. Furthermore, indomethacin markedly enhanced inflammatory responses by increasing proinflammatory cytokines (TNF-α, IL-1β, IL-6), neutrophil infiltration, MPO activity, and activation of the NF-κB/iNOS and p38 MAPK signaling pathways. In addition, activation of the mitochondrial apoptotic pathway, characterized by increased Bax and caspase-3 levels, contributed to epithelial cell loss and mucosal damage. Indomethacin also disrupted gastric mucosal defense by reducing COX-1 activity and PGE₂ levels, while suppressing cytoprotective pathways such as Nrf-2/HO-1. Collectively, these alterations resulted in severe gastric mucosal injury and ulcer formation.

Treatment with syringic acid significantly attenuated these pathological changes in a dose-dependent manner. Syringic acid restored antioxidant defense, activated the Nrf-2/HO-1 signaling pathway, suppressed proinflammatory cytokine production, inhibited neutrophil infiltration, and downregulated NF-κB/iNOS and p38 MAPK signaling pathways. Moreover, syringic acid reduced mitochondrial apoptosis by decreasing Bax and caspase-3 levels and alleviated oxidative DNA damage by reducing 8-OHdG expression. In addition, syringic acid enhanced gastric mucosal defense by increasing COX-1 activity and PGE₂ levels. Molecular docking analyses further confirmed the high binding affinity of indomethacin for COX-1, providing mechanistic insight into its ulcerogenic effect through inhibition of prostaglandin synthesis.

Taken together, these findings indicate that syringic acid exerts significant gastroprotective effects against indomethacin-induced gastric ulcers through its antioxidant, anti-inflammatory, anti-apoptotic, and cytoprotective properties. These protective effects appear to be mediated, at least in part, through activation of the Nrf-2/HO-1 signaling pathway and inhibition of the NF-κB/iNOS and p38 MAPK signaling pathways. Therefore, syringic acid may be considered a promising therapeutic agent for the prevention and treatment of NSAID-induced gastric mucosal injury. However, further experimental and clinical studies are required to confirm these findings and to evaluate the safety and therapeutic potential of syringic acid in humans.

## Data Availability

The datasets generated and/or analyzed during the current study are available from the corresponding author upon reasonable request.
